# Ketogenic Diet: A Review of Composition Diversity, Mechanism of Action and Clinical Application

**DOI:** 10.1155/2024/6666171

**Published:** 2024-10-18

**Authors:** Dominika Malinowska, Małgorzata Żendzian-Piotrowska

**Affiliations:** Medical University of Bialystok, Department of Hygiene, Epidemiology and Ergonomy, ul. Jana Kilińskiego 1, Białystok 15-089, Poland

**Keywords:** clinical application, diabetes, ketogenic diet, mechanism, neurological disease, nutrition support, obesity

## Abstract

The ketogenic diet (KD) is a special high-fat, very low-carbohydrate diet with the amount of protein adjusted to one's requirements. By lowering the supply of carbohydrates, this diet induces a considerable change in metabolism (of protein and fat) and increases the production of ketone bodies. The purpose of this article is to review the diversity of composition, mechanism of action, clinical application and risk associated with the KD. In the last decade, more and more results of the diet's effects on obesity, diabetes and neurological disorders, among other examples have appeared. The beneficial effects of the KD on neurological diseases are related to the reconstruction of myelin sheaths of neurons, reduction of neuron inflammation, decreased production of reactive oxygen species, support of dopamine production, repair of damaged mitochondria and formation of new ones. Minimizing the intake of carbohydrates results in the reduced absorption of simple sugars, thereby decreasing blood glucose levels and fluctuations of glycaemia in diabetes. Studies on obesity indicate an advantage of the KD over other diets in terms of weight loss. This may be due to the upregulation of the biological activity of appetite-controlling hormones, or to decreased lipogenesis, intensified lipolysis and increased metabolic costs of gluconeogenesis. However, it is important to be aware of the side effects of the KD. These include disorders of the digestive system as well as headaches, irritability, fatigue, the occurrence of vitamin and mineral deficiencies and worsened lipid profile. Further studies aimed to determine long-term effects of the KD are required.

## 1. Introduction

The ketogenic diet (KD) has a century-long history of clinical use. Dr Russel Wilder originated the term “ketogenic diet” and designed the diet in 1923 at the Mayo Clinic for the treatment of epilepsy, and it has been used as a treatment option for drug-resistant epilepsy in children ever since. In 1921, Dr Russel Wilder suggested that ketones produced through diet could be as effective as fasting for epileptic seizures [1–4].

The KD has gained considerable interest in recent years because of its promising potential effect on a wide range of diseases [5]. Dietary protocols differ in each case, so it is important to note which modification of the KD was used in the study. The diet has also become considerably popular with regard to weight reduction, particularly due to a reduction in the sense of hunger [6]. Available publications on the subject indicate a potential therapeutic role of the keto diet in treatment of other metabolic conditions [7, 8], such as obesity [9], Type 2 diabetes [10] and neurodegenerative diseases [11–16], and—supportively—in treating certain types of cancer [17–19].

From a dietetic point of view, this diet has significant limitations that involve considerable restrictions of many product groups and, consequently, deficiencies in vitamins and minerals [6].

This review reveals selected opportunities for the use of the KD in the treatment of diseases and outlines the potential mechanisms that determine the therapeutic properties of this diet. Currently, research on the KD is expanding into various areas of health. It is worth noting that although there is a growing body of research suggesting beneficial effects of the KD in these areas, the mechanism of action of the KD still remains unexplained. Understanding this mechanism will help to establish specific recommendations for patients.

## 2. KD

The KD is a special high-fat, very low-carbohydrate diet with the amount of protein adjusted to one's requirements. By lowering the supply of carbohydrates, this diet induces a considerable change in metabolism (of protein and fat) and increases the production of ketone bodies [20, 21].

The primary ketone bodies are *β*-hydroxybutyrate, acetoacetate and acetone. They are produced in the liver (in the mitochondria of hepatocytes) and metabolized in extrahepatic tissues (in the mitochondria of peripheral tissues). On the one hand, accumulated ketone bodies lead to ketoacidosis, protein glycation, oxidative stress, interference with the mechanism of food intake regulation and changes in psychophysical condition [22]; on the other hand, ketone bodies are used as an additional, besides glucose, source of energy. To achieve this state, the amount of carbohydrates must be reduced to a maximum of 10% of the energy value of the diet [23].

### 2.1. Classification

There is no universally accepted classification of KDs that would precisely indicate the percentage share of each macronutrient. To date, several variants of the KD have been developed to enhance adherence to it and simultaneously maintain the effectiveness of the classic version.

The classic KD is a dietary protocol based on consumption of a considerable amount of fats (80%–90% E), with a concomitant low supply of protein (approximately 6%–15% E) and a very low supply of carbohydrates (approximately 5%–10% E) (Figure S1) [24, 25]. In the classic KD, the gram ratio of macronutrients, fat to protein and carbohydrates combined, is 3:1 and 4:1, respectively. At a ratio of 3:1, about 87% of energy comes from fat, while at 4:1—it is 90%. The main source of fats is long-chain triglycerides [26, 27]. There are several methods of initiating the classic KD in order for the body to adapt to and redirect the metabolism to fatty acids as an energy source. The traditional method involves a period of fasting (24–48 h) during hospitalization. Once a high level of ketosis is reached, smaller meals with 90% fat content are introduced. Daily food intake is increased gradually until full-calorie meals are tolerated [28]. Another approach is to gradually increase the KD ratio (the ratio of grams of fats to carbohydrates and proteins combined) to allow the patient to become accustomed to the increasing amount of fat in the diet. This increases daily from 1:1 to 2:1, 3:1 and 4:1 [29]. Alternatively, the diet can be started with target values, that is, a full-calorie diet with a lipid:carbohydrate ratio of 4:1 from Day 1 [30]. All these diet initiation approaches are recognized by the International Ketogenic Diet Study Group [28]. In the classic KD, restrictions on energy and fluid are not necessary. The diet with the composition described above was originally developed to treat drug-resistant epilepsy and is so restrictive that it is recommended for use in therapeutic settings [31].

Such a low intake of carbohydrates is very difficult to maintain (tasteless, difficult to prepare). To facilitate the diet application, several other variants of the classic KD used in clinical practice have been developed. These present similar efficiency to the original form of the diet [32]. They differ in the macronutrient ratio and include a diet incorporating medium-chain fatty acids (MCTs), a modified Atkins diet (MAD) (1:1 or 2:1).

The MCT diet was developed in 1971 as a more acceptable method of nutritional therapy [33]. This diet is dominated by MCTs derived from MCT oil (Figure S1). The medium-chain triglyceride diet (MCTD) guarantees faster absorption of triglycerides into the bloodstream, which results in the formation of more ketone bodies per kilocalorie [34]. Higher efficiency of this process allows for using less fat in the diet and thus allows for more carbohydrate and protein to be consumed, which facilitates long-term maintenance of the diet [33, 34]. In addition, this type of diet improves mitochondrial function [35]. Unfortunately, it may be accompanied by gastrointestinal side effects just as in the case of the classic diet [34].

The MAD is based on a high content of fats relative to other macronutrients, but the ratio of fats varies within much wider boundaries (Figure S1). The ratios of these compounds are not strictly maintained and can range from 1:1 to 4:1. In the MAD, it is assumed that the ratio of fats to carbohydrates and protein combined is 1:1 or 2:1 [36, 37]. During the first month of the diet, carbohydrate intake is limited to 10–15 g/day and then increased to 20 g/day. It does not involve restrictions on protein, fluid or energy intake, which makes it easier to manage the diet [5].

Interest in this type of diet has been growing intensely for many years. According to the data from the PubMed database, there were 605 publications on KD in 2020 and 618 in 2022 (Figure S2). Moreover, trends of creating new forms of the KD can be observed, and there have been reports about the ketogenic Mediterranean diet. There are attempts to promote new models of the KD based on healthy dietary choices [38–41].

The Mediterranean ketogenic diet (MMKD) is based on the Mediterranean diet, which emphasizes healthy fats such as olive oil, lean protein sources like fish and lean meat, and limited consumption of fruits and vegetables (Figure S1) [42].

### 2.2. Mechanism of Action

When following a diet containing a balanced amount of fats and carbohydrates, the substrate for ATP production is glucose, which can undergo glycolysis to produce energy or glycogenesis to produce glycogen [43]. During a KD, glucose is replaced by lipid compounds, which leads to a decrease in glycolysis and increases ketogenesis. The very term “ketogenic diet” is associated with its ability to significantly increase the level of ketone bodies in the body [23].

Ketone bodies are metabolites that are endogenously synthesized during not only adherence to a low-carbohydrate and high-fat diet, but also physiological periods such as short-term fasting or prolonged starvation. Ketogenesis occurs mainly in the liver and—to a lesser extent—in the kidneys [44].

Reducing carbohydrate supply with simultaneous increase in fat intake decreases blood glucose level, which in turn lowers insulin levels. This situation, along with high concentration of adrenaline, leads to the release of free fatty acids (FFAs) from triacylglycerols (TAGs) and glycerol from adipocytes, through the action of hormone-sensitive lipase. The released FFAs are transported to the liver, into the mitochondrial matrix of hepatocytes via carnitine palmitoyltransferase 1 (CPT1), and undergo *β*-oxidation in the mitochondria of hepatocytes, leading to the formation of acetyl-CoA [21, 23].

Under standard conditions, the acetyl-CoA molecule can be utilized in the Krebs cycle through oxaloacetate-mediated incorporation. However, when the carbohydrate supply is low, the hepatic pool of oxaloacetate is used up for the needs of glucose synthesis in the process of gluconeogenesis. The acetyl-CoA molecule is then used to produce acetoacetate (the first ketone body formed, which is also a precursor for other ketone bodies), which is later spontaneously converted, as a result of decarboxylation, to acetone or reduced to *β*-hydroxybutyrate by 3-*β*-hydroxybutyrate dehydrogenase [5, 23]. The produced ketone bodies are released from the liver into the bloodstream. Ketone bodies are primarily catabolized in the mitochondria of extrahepatic tissues to acetyl-CoA, which is oxidized via the TCA pathway (Kerbs cycle, tricarboxylic acid cycle) and releases energy. In particular, this happens in the heart, skeletal muscles and brain and constitutes an alternative energy source. Ketone bodies are also directed to lipogenesis or sterol synthesis pathways, or are excreted in the urine [21]. Acetone, as a volatile substance, is mainly removed from the body via the lungs and kidneys, while acetoacetate and *β*-hydroxybutyrate are transported to extrahepatic tissues where, in the mitochondria, they are used to restore acetyl-CoA molecules, which are then incorporated into the Krebs cycle and ATP production [23].

The mechanism of energy production participated by ketone bodies is more efficient than with the participation of glucose, due to the fact that ketone bodies bypass the glycolytic pathway by entering the Krebs cycle directly [5]. In addition to serving as a source of energy, ketone bodies play other roles, including the role of modulators of inflammation or oxidative stress, or of signalling mediators [21].

The production of ketones is a physiological phenomenon. A state of increased production of ketone bodies is called ketosis. Nutritional ketosis (called “physiological ketosis”) occurs when the blood level of ketone bodies exceeds 0.5 mmol/L [45]. Ketone concentrations in blood can be elevated by such interventions as starvation, KD, prolonged exercise or fasting [46]. In the morning, after an overnight break, the content of ketone bodies is usually higher and amounts to around 0.4 mmol/L. After prolonged workout or 24-h fasting, this level rises to 1 mmol/L [46, 47], while with a diet of low-carbohydrate intake as well as during a KD, the concentration of ketone bodies can rise to more than 5 mmol/L. The level of ketosis depends on the type of diet as well as individual predisposition [48, 49].

## 3. Clinical Application of the KD

There are a number of studies on the use of the KD in various disease entities, mostly related to neurological diseases. In the last decade, there have been an increasing number of results on the effects of the diet on obesity, diabetes, cancer, cardiovascular disease, polycystic ovary syndrome and neurological conditions [9–11, 17, 50, 51].

### 3.1. Neurological Disease

The use of the KD dates back to the early 1920s, when Russell Wilder developed a diet that mimicked the body's metabolic state during starvation and proposed its use in patients with epilepsy. This was when the term “ketogenic diet” was first used [1]. With the subsequent development of antiepileptic drugs, its application was no longer common. It was not until the late 1980s to early 1990s that it was restored and began to be used in the treatment of drug-resistant epilepsy in children [52].

Every therapeutic action taken in diseases of the nervous system is aimed at slowing down or completely stopping the process that leads to the degeneration and death of neurons (neuroprotective actions) [11]. The beneficial effects of the ketogenic dietary therapy on neurological diseases result, inter alia, from the reconstruction of neuronal myelin sheaths, in the reduction of neuron inflammation, reduction of reactive oxygen species (ROS) production, promotion of dopamine production, repair of damaged mitochondria (which affect the disturbed neuronal metabolism in the course of numerous neurological diseases) and formation of new ones [53]. Additionally, the ketogenic dietary therapies provides neurons with an alternative energy source in the form of ketone bodies, which is extremely important because glucose absorption, transport and metabolism are most often impaired in neurological diseases [12, 54]. Many studies suggest the relevance of the KD as an element of adjunctive therapy in the treatment of diseases of the central nervous system, due to the diet's effects on modulating inflammation [55–60], controlling pro-oxidant–antioxidant balance [61–64] or altering the composition of the gut microbiome [65]. This mechanism of action of the KD affects a number of neurological diseases, such as epilepsy, Alzheimer's disease (AD), Parkinson's disease, depression, migraine and multiple sclerosis (MS) [34, 53]. Recent years have also been devoted to the study of the KD as a complementary potential neuroprotective therapy in stroke, headaches, sleep disorders and injuries involving the nerves and the brain [66].

The literature also discusses the use of the KD in psychiatric diseases, such as severe anxiety, depression, active bipolar disorder with psychosis or schizophrenia. Ketones are potentially a neuroprotective factor, primarily due to reduction of inflammation in the body and maintenance of stable blood glucose levels [67–71]. However, these studies are limited to single cases or small groups, and the duration of the diet is short. Nevertheless, the positive effects obtained, such as a significant improvement in symptoms of depression and psychosis, indicate the need for a future randomized controlled trial.

AD is a type of dementia of a multifactorial origin. The main mechanism of the development of this disease is the deposition of *β*-amyloid peptide deposits in the brain as senile plaques and excessive phosphorylation of the tau protein in the form of neurofibrillary tangles. The picture of this disease is characterized by impaired cognitive function and, in a later phase, an inability to function independently [12]. It has been demonstrated that the KD can have a beneficial effect on AD, affecting many bodily processes [72]. Consumption of high-glycaemic-index foods promotes the accumulation of *β*-amyloid in the brain; hence, the KD may have a neuroprotective effect in AD [73, 74]. This correlation was confirmed in 2005 by Van der Auwera et al. on a mouse model of AD. After 43 days of dietary intervention, mice fed the KD showed a 25% reduction in brain levels of *β*-amyloid compared to mice in the control group [74]. Dietary ketosis alone can also exert beneficial effects. In a 2013 study by Kashiwaya et al., the administration of ketone bodies alone to mice with AD also led to a reduction in *β*-amyloid accumulation and improved cognitive functions [75]. So far, there have been several studies on the application of the KD in humans with AD at this point and these were conducted on small groups of patients [13, 76–78]. In 2021, the first randomized controlled trial conducted by Phillips et al. in patients with AD was published. It evaluated the 12-week effect of the KD on the course of the disease. The KD was compared with a standard low-fat diet. Patients from the ketogenic-diet group experienced improvements in cognitive function, daily functioning and the quality of life. In this study, it was also observed that the adverse effects of the diet were mild and that changes in most cardiovascular risk parameters were favourable (decrease in the body weight, BMI and HbA1c, no change in the triglyceride content, and slight increases in the levels of HDL, LDL and total cholesterol from baseline values to Week 12) [79]. Despite problems with adherence to the diet requirements, after 12 weeks of the study, half of the patients chose to continue the diet. The marked change in the quality of life of Alzheimer's patients on the KD may be even greater than with the effect of such drugs as cholinesterase inhibitors, whose effect on the quality of life is varied [80, 81]. Given these results, recommendations for a low-fat diet in AD should be verified and compared to the current research findings [53].

Parkinson's disease is another neurodegenerative disease that is a significant cause of disability worldwide. This is due to the death of dopaminergic neurons in parts of the black matter and is manifested as motor slowing, sensory disturbances, balance disorders and tremor, among other symptoms [81, 82]. The KD in this case acts on chronic nervous system inflammation, excess of ROS, mitochondrial dysfunction, reduced capacity of dopamine production or abnormal cerebral glucose metabolism in the course of the disease [16, 83]. Zhu et al., in their study of 2022 on a rat model of Parkinson's disease, demonstrated the anti-inflammatory effects of the KD [84]. In 2018, Phillips et al. conducted a randomized controlled trial comparing a low-fat diet and a KD in the course of Parkinson's disease. Although improvement of the health status was observed in all patients after 8 weeks, the change was more pronounced in the ketogenic-diet group. Improvement was assessed using the Unified Parkinson's Disease Rating Scale (UPDRS). In first part of the scale, checking nonmotor symptoms, the scale improvement values were higher by 30% points for the KD compared to the low-fat diet. In contrast, the second part, which verifies motor symptoms, showed significant improvement in both groups. Side effects were also observed in both groups. The group applying the low-fat diet reported an increased feeling of hunger, while patients on the KD suffered periodic tremor and/or stiffness [15]. A 2005 study by Vanitallie et al. also revealed changes in UPDRS scores when consuming the KD. Following the diet by five patients for 28 days resulted in a mean total decrease in UPDRS scores of 43.4%, with additional improvements in energy levels, mood, posture and gait in terms of a better sense of balance and resting tremor [85]. In their study of 2022, Tidman, White and White demonstrated a beneficial effect of the KD applied for 12 weeks to Parkinson's disease patients. Improvement was observed in the Parkinson Anxiety Scale (PAS) results, also in the first part of the UPDRS, but also in such parameters as body weight, glycated haemoglobin and fasting insulin, as well as HDL cholesterol and triglycerides, among other parameters. In contrast, no significant changes were observed in depressive symptoms on the Centre for Epidemiologic Studies Depression Scale Revised (CESD-R-20) [86]. Considering the potential of the KD in the treatment of Parkinson's disease as well as the wide range of effects of this diet on many aspects, further research related to the topic appears to be necessary [53]. MS is a chronic, progressive, inflammatory and degenerative disease of the central nervous system. It is characterized by the formation of multifocal and diffuse inflammatory demyelinating lesions that lead to damage and loss of axons in the brain and spinal cord [87]. MS is recognized as a disease resulting from an autoimmune process in which immune activity is directed against myelin antigens in the central nervous system [88]. The clinical manifestation of the disease is associated with a range of neurological abnormalities such as problems with mobility, sensation, vision, sphincter control, fatigue, mood and cognitive dysfunction, leading to progressive disability. Symptoms depend on the location and size of the demyelinating plaques [89]. Research into the role of the KD in MS is in the observational phase. The KD has immunomodulatory properties that may benefit people with MS [90]. Ketones may have a protective effect on myelin by influencing repair and regeneration processes in the central nervous system [91]. In addition, the KD has the potential to reduce inflammation, which contributes to the progressive loss of myelin in MS patients [92, 93]. Zyla-Jackson's et al. study of the KD in people with MS confirms that this diet, enriched with MCTs, omega-3 fatty acids and fibre, has anti-inflammatory effects and alleviates autoimmune-induced demyelinating visual and motor deficits [94]. A 2019 study by Lee and Choi suggests that a KD improves cognitive function and reduces fatigue (one of the main symptoms of the disease) in people with MS. However, the authors stress that more research is needed to confirm these effects [95]. A 2020 meta-analysis by Kim et al. suggests that the KD affects the expression of genes associated with the immune response to MS-related processes. The authors highlight that there is evidence for a beneficial effect of the KD on neurological function and quality of life in patients with MS [96]. The KD in MS patients in the Choi et al. study showed clinically significant improvements in the Health-Related Quality of Life (HRQOL) scale, which includes overall quality of life, and physical and mental health composite, after 3 months [93]. In addition, KDs have been shown not to worsen the biomarkers of neurodegeneration in patients with MS [97]. Brenton et al. in the study documented half as many reports of fatigue and depression among patients. In patients, the diet reduced fat mass, which has the effect of reducing inflammation in the body. An increase in the physical and mental health quality of life index was also seen during the diet [98]. However, Lee et al. study shows that people on an MCT-based KD did not achieve significant clinical improvement despite achieving nutritional ketosis [99]. Preliminary data suggest that the KD is safe, feasible and potentially effective in the treatment of MS, but further research is needed [93, 100]. However, it should be emphasized that although these results are promising, further studies, including randomized placebo-controlled trials, are needed to more clearly assess the efficacy of the KD in the treatment of MS. In addition, the long-term effects of the KD in MS are unknown, and side effects require additional attention (e.g. side effects may be more problematic in MS patients who already have gastrointestinal problems). Individual patient differences also need to be considered.

Analysing the available scientific data, it can be concluded that the KD is indeed a promising nutritional model for the treatment of the neurological diseases described above [53]. However, it should be noted that most of the said studies were conducted on animals, while clinical trials involved a small group of patients and the duration of the research was relatively short. Elder people with neurodegenerative diseases are usually at risk of malnutrition, so applying of a diet that reduces appetite and causes gastrointestinal distress may be counterproductive. Further research is required on the subject—an in-depth analysis of the impact of the KD on the treatment of neurological diseases, investigation of the long-term effect of the diet (long-term randomized clinical trial), and considering this diet as one of treatment choices in neurological diseases other than those mentioned in this work [23]. Future research may make the KD a dietary regimen in the clinical therapy of neurological patients, thus improving the quality and prolonging the life of people suffering from neurological diseases (currently the incidence of neurological diseases is on an upward trend) [53].

### 3.2. Diabetes

Type 2 diabetes is the most common type of diabetes and is characterized by high morbidity and mortality rates worldwide due to multiple complications caused by it [101]. Despite the application of pharmacotherapy, which can reduce blood glucose fluctuations, effective and recommended forms of treatment include nonpharmacological intervention, that is, lifestyle changes, in particular regarding one's diet [102]. As of today, according to the Polish Diabetological Association (PTD), several dietary strategies can be used to treat diabetes. They are based on minimally processed foods, require the consumption of considerable amount of non-starchy vegetables and minimize added sugars and refined cereals—these include the Mediterranean diet, the DASH diet, the Flexitarian diet and the plant-based diet [103]. However, a recent report by the American Diabetes Association (ADA) recommends a low-carbohydrate diet as an appropriate dietary approach in patients with diabetes or prediabetes. According to this report, following a low-carbohydrate diet in patients has a positive effect on reducing blood glucose level and HbA1c. It should be noted that these recommendations do not refer directly to the KD but to low-carbohydrate diets in general [104].

Research on the effects of the KD on diabetes has mostly focussed on Type 2 diabetes, while for Type 1 diabetes, the number of studies is low. This is most likely due to the fact that there is an increased risk of hypoglycaemia and ketoacidosis in these patients [105]. However, there has been research on the narrow scope of the effect of the KD on Type 1 diabetes [106–110]. Leow et al. conducted a long-term observation-based study of approximately 3 years in a small group of adults with Type 1 diabetes. The KD was shown to be associated with normal HbA1c levels and little glycaemic variability, but cases of dyslipidaemia and a high number of hypoglycaemic episodes also occurred when using this diet [107]. Another concern is the studies that demonstrate the cases of accelerated ketoacidosis in people with Type 1 diabetes when on the KD [111]. An important contraindication to the use of the keto diet in the treatment of diabetes is also the administration of drugs that contain SGLT-2 inhibitors due to the increased risk of ketoacidosis in such patients. Therefore, considerable caution should be exercised in patients with Type 1 diabetes who wish to follow the KD [105].

Chronic hyperglycaemia, which is associated with uncontrolled diabetes, leads to many serious, often irreversible, changes in the body. The causes of Type 2 diabetes are genetic and environmental factors, including overweight, obesity and sedentary lifestyle. Recently, an increasing interest has been observed in the KD as a form of treatment of Type 2 diabetes with concomitant obesity [112]. There are data confirming that the use of the KD improves a patient's condition in the course of hyperglycaemia. This consists in a reduction in circulating blood glucose level and an increase in tissue sensitivity to insulin [113]. The beneficial effect of the KD in diabetes is related to the characteristics of this diet [105]. Minimizing the intake of carbohydrates entails reducing the absorption of simple sugars, thereby decreasing blood glucose concentration and glycaemic fluctuations [114].

A 2-year, open-label, nonrandomised, controlled study conducted by Athinarayanan et al. compared the use of a standard diet versus the KD among 349 participants. At the end of the study, in the KD group, there was a decrease in HbA1c levels, while in those on the standard diet, the content of HbA1c increased [115]. A similar study by Iqbal et al. demonstrated no significant difference in HbA1c levels after 2 years on either of the two diets: low-fat and low-carbohydrate [116]. In the 2021 meta-analysis by Goldenberg et al., the efficacy of low- and very low-carbohydrate diets was tested in patients with Type 2 diabetes and confirmed the benefits of the KD in the areas of weight loss, control of glycaemia and sensitivity to insulin, which were evident in the short term of applying the diet. After 12 months, however, the benefits began to fade. In addition, long-term adherence to such a diet may be connected with difficulties in following it (due to a very restrictive eating pattern) [117]. The above studies prove that a shorter period of using the KD is associated with a significant improvement in results related to glycaemia control, while in the long term (despite the existing examples of improvement), it is less pronounced.

In a 2017 meta-analysis, Meng et al. evaluated the effect of low-carbohydrate diets, including the KD, and compared them with normal- and high-carbohydrate diets in people with Type 2 diabetes. They analysed 734 subjects in total and showed that low-carbohydrate diets (including the KD) significantly reduced HbA1c levels, in addition to significantly reducing cardiovascular risk factors, including blood triglycerides and increased HDL content, while LDL levels and total cholesterol remained unchanged [118]. Studies by Dyson et al. also confirm the efficiency of very low-carbohydrate diets (< 40 g and < 30 g of carbohydrates in the diet) in the treatment of diabetes [119, 120].

One of the most common causes of Type 2 diabetes is overweight and obesity. Obesity is widespread among Type 2 diabetics. This condition is associated with chronic inflammation (including endoplasmic reticulum stress and hyperinsulinemia) [114]. Another important factor accompanying the KD is weight loss, which is an effective intervention in weakening tissue resistance to insulin [105, 114]. The results of a 2022 meta-analysis by Zhou et al. suggest that the use of the KD in overweight Type 2 diabetic patients has significant benefits in terms of body weight (reduction in the body weight and waist circumference), glycaemic control (reduction in HbA1c concentration) and improvement in the lipid profile (reduction in triglyceride and increase in HDL levels) [114].

There is information in the literature about the beneficial effects of a low-carbohydrate diet that does not induce a state of ketosis. Therefore, it remains to be examined whether the advantageous effect of the KD in the treatment of diabetes is a direct result of its use and the metabolic changes that occur during its use, or whether it is only due to weight reduction [114]. It should also be borne in mind that there is a risk of worsening of the lipid profile due to adhering to high-fat and low-carbohydrate diets [107, 121].

It is noteworthy that a growing body of evidence indicates a strong effect of the KD in insulin resistance [122, 123]. Even a low increase in peripheral blood ketosis, induced with the KD, can reduce stress connected with hyperinsulinemia, improve sensitivity to peripheral insulin, lower external demand for insulin and slow down insulin secretion. These mechanisms, in turn, may thus improve the glycaemic profile and alleviate insulin resistance [124]. Additionally, ketone bodies can elevate intracellular glucose levels and produce metabolic effects that are similar to insulin. At the same time, the insulin signalling pathway is not activated, which allows for a therapeutic effect of mild ketosis in states of insulin resistance [122]. According to a meta-analysis of eight studies from 2022 on overweight patients with Type 2 diabetes mellitus (T2DM), the KD had a significantly beneficial effect on weight loss and contributed to the reduction in the waist circumference, decreasing the levels of glycated haemoglobin and triglycerides, and increasing the content of high-density lipoproteins. Zhou et al. therefore suggest a great potential of this diet in prediabetic conditions to prevent the development of the disease.

However, it is important to remember that, according to recommendations by the ADA, there is no one correct dietary approach in patients with diabetes. Diet selection should be individual, taking into account the latest indications and personal preferences. As the long-term effects of the KD in patients with diabetes are still unknown, people who decide to follow this diet should be under constant medical supervision and should undergo tests for, inter alia, kidney disease [23].

### 3.3. Obesity

The KD may be one of the solutions for the treatment of obesity due to numerous studies confirming its safety and efficacy. Bueno et al. performed a randomized meta-analysis of 13 studies. The researchers compared the use of a low-energy KD (< 50 g of carbohydrates per day) and an energy-restricted low-fat diet (< 30% of energy from fat) in overweight and obese individuals for over 12 months. The results of the meta-analysis showed that, in the long term, the low-energy KD had better effects on weight loss, reducing the level of triglycerides and diastolic blood pressure and leading to a greater increase in HDL cholesterol and also a greater increase in LDL cholesterol [125]. Nordmann et al., in their meta-analysis of 2006, compared the KD (< 60 g of carbohydrates per day) with a low-fat diet without energy restriction. After 6 months of applying the diets, a greater weight reduction was observed in those following the KD, whereas after 1 year of using these diets, no significant differences were found among participants [126]. Hu et al., in a 2012 meta-analysis of 23 studies, compared a low-carbohydrate diet (without isolating the KD, with carbohydrate content ranging from 4% to 45%) and a low-fat diet (≤ 30% energy from fats) and observed no differences between the diets with regard to weight loss and waist circumference reduction [127]. Sackner-Bernstein, Kanter and Kaul performed a meta-analysis investigating 17 studies comparing low-carbohydrate and low-fat interventions in overweight and obese individuals. In both groups, the low-carbohydrate group and the low-fat group, energy intake was similar. Both diets showed a significant reduction in the body weight and decreased predicted risk of coronary events. However, the low-carbohydrate group presented greater statistically significant improvement in both aspects studied [128].

The KD and weight reduction has been a popular topic in recent years. Studies suggest the superiority of the KD over other diets for weight loss. However, due to the lack of long-term studies on using the diet to assess its long-term effects, the debate on the validity of this diet remains open [23]. According to numerous meta-analyses and reviews regarding the KD and its effect on weight control, such process as dietary ketosis appears beneficial [125, 129–131]. What exactly influences the weight loss, that is, the mechanisms responsible for it during the KD, have not been comprehensively elucidated yet. There are several hypotheses to explain this phenomenon, including that it may be due to the regulation of the biological activity of appetite-controlling hormones [132] or to decreased lipogenesis, boosted lipolysis and increased metabolic costs of gluconeogenesis [133]. It certainly depends on the concurrence of multiple processes, with appetite reduction in the course of this diet being a significant action [134].

Some studies available demonstrate spontaneous reduction in energy intake, while on the KD, out of one's will, leading to carbohydrate intake reduction to 5%–10% of the dietary energy content [134–136]. Appetite suppression is likely to result from, inter alia, changes in hormonal and neuronal anorexigenic and orexigenic factors, an increase in the level of circulating FFA, and from the direct effect of ketone bodies [134]. During the KD, there is a noticeable increase in the secretion of cholecystokinin (an anorexigenic factor), which directly affects the satiety control centre in the hypothalamus and decreases the release of ghrelin (hunger hormone—enhances food intake) [137]. Appetite suppression may also be mediated by leptin, which, when it binds to its receptor in the hypothalamus, reduces the action of orexigenic compounds and increases the release of anorexigenic compounds [138]. However, data related to studies on leptin are inconsistent due to the fact that under dietary ketosis conditions, animal studies show an increase in leptin concentrations [139–142], while humans demonstrate a decrease [143–146] or no changes [147]. It has also been observed that in the course of the KD, the total daily energy expenditure of the body is slightly increased [148]. With a reduced amount of carbohydrates in the diet, the process of gluconeogenesis is intensified, resulting in an increase in daily energy requirement by 400–600 kcal [149]. Another proposed mechanism to accompany the KD is a change in resting energy expenditure. In 2012, Ebbeling et al. examined the effect of diet composition on energy expenditure during weight maintenance and weight loss. Under isoenergetic conditions, they noted that a carbohydrate-restricted diet was better for maintaining basal metabolism in comparison with a low-fat diet. Compared to baseline before the diet was introduced, the decrease in resting energy expenditure while on the low-fat diet averaged 205 kcal/d, while on the low-carbohydrate diet, it was 138 kcal/d [150]. Individual characteristics are more and more often suggested in terms of the effectiveness of diets. In a 2018 study, Brouns observed that the KD (a high-fat diet accompanies increased production of ketone bodies) is more effective for weight loss in prediabetic and diabetic individuals, while a low-fat diet (a relatively high-carbohydrate diet) is more recommended for individuals with normal glucose levels [151].

Further research on long-term adherence to the diet in question and its practicality is still needed. When testing the effectiveness of the KD in reducing body weight, it is important to look at the diet duration. The rapid initial weight loss with this diet can be attributed in part to water loss [152]. A study by Mansoor et al. (11 randomised controlled trials lasting 6 months) comparing low-carbohydrate and low-fat diets demonstrated that participants following low-carbohydrate diets lost 2.17 kg more than those adhering to low-fat diets [153]. However, when looking at studies with a longer duration of adherence to a diet, the difference in weight loss is much smaller. Bueno et al. (13 randomized controlled trials lasting a minimum of 12 months) also compared a very low-carbohydrate (ketogenic) diet and a low-fat diet, showing a weight reduction of 0.91 kg greater in the ketogenic-diet group compared to the low-fat-diet group [125]. One has to face a number of difficulties when comparing the effects of different diets in the treatment of obesity. There are no universally accepted protocols available for each type of diet that specify macronutrient ratios, which limits the ability to compare results from different studies. Problems with the KD often include the lack of monitoring the subjects' ketosis status, making this diet indistinguishable from low-carbohydrate diets. The best approach to compare the effectiveness of diets is to compare isoenergetic diets. However, with the KD, because of the reduction in appetite, it may make it easier for the respondent to adhere to the energy regime of the diet. Additionally, with such a high-fat diet, excess amino acid intake—even with appropriately restricted carbohydrates—may prevent achieving the state of ketosis. Some amino acids are glucogenic and lead to unintentional glucose production [154]. In addition, the ratio of macronutrients consumed to achieve a state of ketosis may itself be individual in nature [155]. The potential of the KD is also being investigated in other diseases, such as cancer [17–19], cardiovascular disease [51, 126, 153], metabolic syndrome [40, 128] and PCOS [50, 156]. However, in addition to the numerous beneficial properties of the diet, it is also worth paying attention to what effects such a restrictive diet may have on the body.

### 3.4. KD in Context of COVID-19

Recently, researchers have begun to highlight the potential benefits of the KD in the context of infectious diseases, with a particular focus on the COVID-19 pandemic [157–159]. KD potentially affects the immune response to respiratory viruses such as SARS-CoV-2. Studies suggest that ketones affect immunomodulatory processes: They regulate the activity of macrophages, T lymphocytes or pro-inflammatory cytokines [159–161]. The KD also affects autophagy processes involved in the removal of damaged cells and viruses from infected cells. This improves general immunity [162]. The results of Sukkar et al. demonstrate the therapeutic role of eucaloric ketogenic diet (EKD) through immunomodulation in the clinical management of COVID-19. A retrospective pilot study provides valuable preliminary information on the reduction of mortality and ICU admissions in patients [163]. Karagiannis et al. showed that dietary glucose restriction administered to HFKD mice largely destroyed Type 2 innate lymphoid cells (ILCs-2) residing in the lungs and reduced airway inflammation [160]. KD is also associated with a reduction in inflammation, helping to control the effects of viral infection (an excessive inflammatory response can contribute to the pathology associated with viral infection) [164]. In addition, a KD alters cellular metabolism, which affects the ability of cells to replicate the virus. Under certain conditions where cells are glucose-dependent, a reduction in glucose availability may limit viral replication and reduce the severity of disease symptoms [157, 165, 166]. Intervention with a KD may prove effective both in the prevention and during COVID-19 infection, particularly in patients with existing medical conditions such as obesity or diabetes. Beneficial effects include the reduction of inflammation (chronic inflammation is associated with a more severe course of many diseases, including COVID-19), regulation of glucose metabolism, improvement of insulin sensitivity in diabetics and weight loss in obesity [167–169]. Da Eira et al. showed that therapies such as MSKD (medically supervised KD), which induce rapid and significant weight loss, can have a beneficial effect on hospitalization rates and COVID-19 severity in patients with T2DM. In addition to vaccination, and in line with public health recommendations, MSKD may be an effective preventive strategy to reduce the severity of future COVID-19 in people with T2DM and obesity [167, 170]. In addition to the above, current research describes the effect of the KD on the regulation of cytokines, particularly those associated with the so-called “cytokine storm” observed in some patients with COVID-19 [166, 171]. Severe coronavirus 19 (COVID-19) disease is characterized by cytokine storm syndrome (CSS), which is associated with excessive macrophage activation. The KD reduces the severity of the cytokine storm in SARS-CoV2 infection through immunomodulation of M1 macrophages [166]. In addition, ketone catabolism is an important source of ATP when the cytokine storm leads to a block in mitochondrial oxidation of carbohydrate catabolites [172]. The introduction of a KD in conjunction with a potential or existing COVID-19 infection requires caution and an individualised approach. A doctor should be consulted before starting a KD. In patients with existing medical conditions such as diabetes or obesity, which are risk factors for more severe disease, the approach to the diet should be tailored to their specific medical condition. Health monitoring is important, including regular measurement of blood pressure, blood glucose and other health parameters. A KD can lead to deficiencies in certain nutrients, so it is important to consume a variety of foods, especially those that boost the immune system. If the diet is deficient, supplementation should be considered.

## 4. Limitations of Scientific Studies on the Effects of the KD on Health Problems

Most studies have small patient populations, and there are few long-term, well-designed, randomized clinical trials in this area. In addition, the range of daily dietary carbohydrate reduction in the reported trials varies, and there is often a lack of information on the protein content. The classic KD is strict and difficult to follow, so we find many variations of it. As a result, there is no single dietary protocol for the diet. In addition, studies are often based on participants reporting the composition of their meals, which is not always accompanied by blood or urine ketone levels. As a result, there is no certainty that they have reached a state of ketosis, which makes it impossible to clearly identify the type of diet and may lead to misinterpretation of the results. KD has a pleiotropic effect on the body and can therefore induce many changes in the body that may go undetected because they are beyond the scope of the analysis planned in the study or the capabilities of the test methods used.

The KD can have various side effects, and not everyone is physically or mentally ready for it. Therefore, the ethics of the research must include strict criteria for the selection of participants and appropriate monitoring of their health status. We also need to consider the long-term effects of the KD. Does the research include the monitoring of participants' health after the diet has ended? If not, how can we be sure that such a diet does not carry a risk of serious health consequences? KD may affect different groups of people differently, depending on their age, gender or pre-existing medical conditions. By looking at different age groups and ethnicities, we can get more comprehensive and realistic results.

## 5. Risks Associated With the KD

The KD is connected with health risks; therefore, this dietary model requires medical supervision. The high-fat, low-carbohydrate diet leads to a number of side effects.

The side effects that occur during the initial period of the diet include disorders of the digestive system, that is, nausea, diarrhoea, vomiting, constipation and abdominal pain. These are a consequence of excessive fat intake [11, 173]. Initially, in addition to gastrointestinal complaints, there are also headaches, irritability, fatigue and dehydration [173, 174]. The diet may also lead to hypoglycaemia, acidosis, pancreatitis or hepatitis, as well as hypercholesterolaemia, hypertriglyceridaemia, hyperuricaemia, hypomagnesaemia or hyponatraemia [11, 175]. Dietary ketosis leads to a decrease in appetite during the diet. It has a hormonal and neuronal effect on the hunger and satiety centres. In addition, the monotonous, unattractive taste of the diet reinforces food aversion and reduces the motivation to maintain the diet—particularly in the case of the classic KD [5, 23]. When treating obesity, this is a desirable effect of the diet, which determines adherence to its energy content [176]. However, in malnourished patients, especially those with cancer or neurodegenerative diseases, the said effect is undesirable [11, 176, 177].

Long-term adverse effects of the KD may include the occurrence of vitamin and mineral deficiencies, which is caused by high dietary restrictions. However, this can be prevented if proper selection of products in the diet is ensured. A 2019 study by Taylor et al. on a small group of patients showed that people who are offered support while on the diet can have a nutrient-rich KD [178]. Despite this fact, the KD should be accompanied by supplementation with sugar-free preparations of water-soluble vitamins of group B, which cannot be supplied by the diet. It is also practicable to additionally supplement omega-3 fatty acids, minerals (selenium, zinc, calcium) and carnitine during the diet [176, 179].

Another risk associated with application of the KD is changed lipid profile [180]. In 2021, Burén et al. examined the effects of a 4-week KD (rich in saturated fatty acids) in healthy, young, normal-weight women on the lipid profile. The KD in each woman who completed the study led to increased LDL cholesterol, total cholesterol and apolipoprotein B-100 (ApoB) [181]. In a randomised controlled trial from 2018, the effects of a low-fat diet and a high-fat diet in overweight and obese people were compared, and the effect on lipid profile, among other parameters, was examined. A more pronounced reduction in total cholesterol and LDL cholesterol levels was found following a low-fat diet compared to a high-fat one [182]. A randomized, crossover, 2022 intervention study assessed the effect of the KD versus the Mediterranean diet in adults with prediabetes or Type 2 diabetes. The KD increased LDL cholesterol in the participants by 10% but also decreased triglycerides by 16% and increased HDL cholesterol by 11% [183]. A meta-analysis of 14 studies from 2020 focussed on the effectiveness of the KD in overweight or obese patients as well as those with or without Type 2 diabetes. It demonstrated that the KD was more effective in improving metabolic parameters (controlling glycaemia, body weight and lipid content) in overweight or obese patients, in particular those with associated diabetes, compared to low-fat diets. The KD led to significant weight loss and improvement in lipid parameters by reducing the levels of triglycerides and increasing HDL cholesterol, but it was also associated with elevated values of LDL cholesterol—a relatively greater increase was noted among patients without diabetes [184]. On the other hand, in a 2002 study by Sharman, a 6-week KD in normal-weight men with normolipidemia did not affect total cholesterol and LDL cholesterol but increased HDL cholesterol [185]. In addition, a survey (on the KD) conducted by Patel et al. in 2010, conducted among parents of children with drug-resistant epilepsy, revealed that the blood lipid profile normalizes after the discontinuation of the therapy (the median time from discontinuation of the KD was 6 years), and no atherosclerosis, cardiomyopathy or coronary artery disease was noted in the subjects [186].

A 2003 study by Miętkiewska et al. shows that the KD offers low calcium and dietary fibre content. In the long term, inadequate supply of calcium may be associated with an increased risk of reducing bone mineral density. On the other hand, dietary fibre deficiency correlates with an increased risk of colon cancer [6, 187].

Other adverse effects of the KD include optic neuropathy, anaemia, renal calculi and cardiomyopathy [11]. Ketoacidosis is also a risk when following a KD for Type 1 diabetes, alcoholism but can also occur when following the diet while breastfeeding [188–190].

Conducting studies to determine the long-term effects of the KD is sorely needed. The KD is becoming increasingly popular. Before introducing a diet that is so different from the basal diet, tests should be necessary to rule out metabolic diseases such as porphyria, primary carnitine deficiency, pyruvate carboxylase deficiency, defects related to fatty acid oxidation or mitochondrial disorders [191].

## 6. Summary

The KD is a high-fat, very low-carbohydrate diet providing an adequate amount of protein. This diet, by reducing the supply of carbohydrates, induces a considerable change in metabolism (of proteins and fats) and boosts the production of ketone bodies. During its use, glucose is replaced by lipid compounds, which leads to a decrease in the intensity of glycolysis and intensifies ketogenesis. To date, several variants of the KD have been developed. In addition to the classic KD, we can distinguish the MCT diet and a MAD.

There are a number of studies on the use of the KD in various disease entities. In the last decade, more and more results of the diet's effects on obesity, diabetes and neurological disorders, among other examples have appeared. The beneficial effects of the KD on neurological diseases are related to reconstruction of myelin sheaths of neurons, reduction of neuron inflammation, decreased production of ROS, support of dopamine production, repair of damaged mitochondria (which affect the disturbed metabolism of neurons in many neurological diseases) and formation of new ones. In addition, the KD provides an alternative source of energy for neurons in the form of ketone bodies, which is extremely important because glucose absorption, transport and metabolism are most often impaired in the course of neurological diseases. The beneficial effect of the KD in diabetes is due to the characteristics of this diet. Minimizing the intake of carbohydrates results in reduced absorption of simple sugars, thereby decreasing blood glucose levels and fluctuations of glycaemia. On the other hand, studies on obesity indicate an advantage of the KD over other diets in terms of weight loss. This may be due to the upregulation of the biological activity of appetite-controlling hormones, or to decreased lipogenesis, intensified lipolysis and increased metabolic costs of gluconeogenesis. It certainly depends on the interplay of numerous processes, with an important action of this diet being the reduction of appetite.

However, it is important to be aware of the side effects of the KD. In the initial period of using this diet, these include disorders of the digestive system as well as headaches, irritability and fatigue. Long-term adverse effects of the KD include the occurrence of vitamin and mineral deficiencies and worsened lipid profile. The KD is becoming increasingly popular. Further studies aimed to determine long-term effects of the KD are required.

## Data Availability

All relevant data are included within the article.

## Supporting Information

Supporting Information Figure S1: Percentage of individual nutrients in selected types of KDs. ND: normal/habitual diet; CKD: classic KD; MCTD: medium-chain fatty acid–based diet; MAD: modified Atkins diet; MMKD: Mediterranean KD. Figure S2: Number of publications for the search of term “ketogenic diet” in PubMed from 1 January 1931 to 4 May 2023. Search data: 4 May 2023 (PubMed articles from before 1931 were not included due to lack of availability on PubMed).
